# Humidity Sensing by Chitosan-Coated Fibre Bragg Gratings (FBG)

**DOI:** 10.3390/s21103348

**Published:** 2021-05-12

**Authors:** Rosaria D’Amato, Andrea Polimadei, Gaetano Terranova, Michele Arturo Caponero

**Affiliations:** Photonics Micro and Nanostructures Laboratory, Fusion and Technologies for Nuclear Safety and Security Department, FSN-TECFIS-MNF, ENEA C.R. Frascati, Via E. Fermi, 45, 00044 Frascati, RM, Italy; rosaria.damato@enea.it (R.D.); andrea.polimadei@enea.it (A.P.); gaetano.terranova@enea.it (G.T.)

**Keywords:** FBG, chitosan, RH sensor

## Abstract

In this work, we report novel relative humidity sensors realized by functionalising fibre Bragg gratings with chitosan, a moisture-sensitive biopolymer never used before for this kind of fibre optic sensor. The swelling capacity of chitosan is fundamental to the sensing mechanism. Different samples were fabricated, testing the influence of coating design and deposition procedure on sensor performance. The sensitivity of the sensors was measured in an airtight humidity-controlled chamber using saturated chemical salt solutions. The best result in terms of sensitivity was obtained for a sensor produced on filter paper substrate. Tests for each design were performed in the environment, lasted several days, and all designs were independently re-tested at different seasons of the year. The produced sensors closely followed the ambient humidity variation common to the 24-h circadian cycle.

## 1. Introduction

Humidity is an essential parameter monitored in industrial and agricultural applications such as manufacturing and storage of electronic components, food storage, air conditioning, environmental control, pharmaceutical production, and medical procedures [1].

Humidity refers to the content of water vapour in the air or other gases. Its measurement is usually given in terms of Relative Humidity (RH), which is the ratio, expressed in percentage, of water vapour pressure present in the air (or other gases), to the saturation water vapour pressure, at a particular temperature and pressure. Another important parameter related to humidity is the dew point, which is the temperature at which the air (or other gases) begins to condense at a given pressure.

Over the last few decades, optical fibre technology has significantly evolved and a wide range of physical, chemical, and biological sensors based on this technology have been developed. Many different parameters, such as displacement, temperature, pressure, refractive index, current, electric field, magnetic field, and humidity can be measured using fibre optic sensors. Moreover, fibre optic sensors offer several advantageous features, such as compactness, immunity to external electromagnetic interference, corrosion resistance, multiplexing, and easy cabling for remote sensing [2].

A wide range of fibre optic humidity sensors is reported in the literature [3,4,5,6,7,8,9,10,11]. Sensors providing small error and fast response, and for which fabrication methods and interrogation systems are simple and less expensive, are reported in [12,13,14]. Two common measuring principles are attenuation and spectroscopy. The former is based on intensity modulation. The light propagating through the optical fibre is absorbed or scattered as humidity changes. The latter is based on diffraction. A diffraction grating stays in the optical fibre and the diffracted wavelength is affected by humidity changes. 

Fibre Bragg grating (FBG) sensors, for which the working principle and main features are presented further below in this paper, can be made by the deposition of a hygroscopic swelling material on the fibre. The diffracted wavelength changes as a consequence of the swelling induced by humidity. A specific advantage of FBG technology comes from the possibility to use it for the production of sensors for different parameters (humidity, temperature, strain, …), and to have all of them connected in series at the same channel of an FBG interrogation system [2]. In this paper, the authors propose their contribution to the choice of an effective hygroscopic swelling material for the production of humidity sensors based on FBG technology.

Several coating materials have been investigated to fabricate RH sensors based on FBG technology, such as polyimide, di-ureasil, polyvinylalcohol, poly(methylmethacrylate), graphene oxide, and carbon nanotubes [15,16,17,18,19,20,21,22,23]. The authors tested biopolymers, such as agar and agarose, for medical applications [24,25,26,27], and to monitor humidity in the environment and moisture in stones for the conservation of cultural heritage [28,29].

In this work, chitosan is tested as a hygroscopic polymer to fabricate FBG RH sensors, and the influence of coating design and deposition procedure on sensor performance is investigated. Chitosan, which is one of the most abundant polysaccharides in nature, can be easily turned into a hydrogel of high elasticity when dispersed in dilute organic acids, such as acetic acid. Owing to its good film-forming ability, non-toxicity, biocompatibility, and favourable chemical resistance property, chitosan is widely explored in environmental and biochemical fields for drug delivery, food packaging, and biomedicine [30]. Its abundant amino and hydroxyl groups enhance reversible adsorption and desorption of water vapour molecules from the gas phase through hydrogen bond formation [31]. As a result, chitosan swells in the presence of water vapour, thus making it a good candidate as a coating material for FBG RH sensors. However, to the best knowledge of the authors, no papers report the use of chitosan for FBG RH sensing. The use of chitosan is only reported for optical sensors based on technologies different from FBG, such as Fabry–Pérot interferometers [31,32], Long Period Grating [33], and Michelson interferometers [34].

The authors already tested a blend of agar and chitosan as a hygroscopic swelling material for FBG RH sensors, achieving higher sensitivity and a better production control than when using pure agar [29]. In this work, the authors investigate the features of FBG RH sensors based on chitosan coating produced with a few different designs.

## 2. Materials and Methods

### 2.1. FBG Working Principle

A FBG is a phase grating, typically a short segment of single-mode optical fibre with a photo-induced periodically modulated refractive index in the core of the fibre [35]. When broadband light reaches the grating, light is reflected and its spectrum has a peak at the Bragg wavelength λ_B_,

λ_B_ = 2Λn_eff_ (1)

where n_eff_ is the effective refractive index at the grating and Λ is the grating pitch. The dependences of both n_eff_ and Λ on strain and temperature allow the use of FBGs to directly measure those two parameters. By suitable thermal or mechanical transducers, FBGs can be used to indirectly measure various other parameters. A recent review of the growing number of sensing applications of FBGs is given in [36].

For a Bragg grating produced in a single-mode optical fibre subjected only to axial strain, the following simplified equation can be used [37]:

(2) $$\frac{\Delta \lambda_{B}}{\lambda_{B}}=\left(1-P_{e}\right)\varepsilon_{z}+\left(\left(1-P_{e}\right)\alpha +\xi \right)\Delta T$$

where P_e_ represents the effective strain-optic coefficient, in which the radial strain is accounted for the Poisson effect. Equation (2) shows the linear combination of the mechanical and the thermal response of the FBG: ε_z_ is the axial strain; α is the coefficient of thermal expansion of the fibre; ξ is the thermo-optic coefficient; ΔΤ is the temperature change.

For a polymer-coated FBG RH sensor isolated from external mechanical disturbances, the response to RH and temperature is given by

(3) $$\frac{\Delta \lambda_{B}}{\lambda_{B}}=\left(1-P_{e}\right)\varepsilon_{\mathrm{RH}}+\left(1-P_{e}\right)\varepsilon_{T}+\xi \Delta T$$

where the axial strain in the fibre is separated into the RH-induced strain ε_RH_, and the temperature-induced strain ε_T_. These strains arise from the hygroscopic and thermal expansions of the materials constituting the sensor and can be determined using a one-dimensional model of the sensor in which the radial strain is neglected.

To show explicitly the dependence of Δλ_B_ on RH and temperature variations, the expression (3) can be written as

(4) $$\frac{\Delta \lambda_{B}}{\lambda_{B}}=S_{\mathrm{RH}}\mathrm{RH}+S_{T}\Delta T$$

where S_RH_ and S_T_ are the RH and temperature sensitivity coefficients, respectively.

### 2.2. Fabrication of the Samples

Commercial FBGs (Broptics Technology Inc., smf fibre with acrylate coating, 1 cm long grating with acrylate recoating), were used to produce the samples. The FBGs were cleaned with ethanol, and the optical fibres were laid and stretched either in a mould or on a plane substrate; samples were thus produced by depositing chitosan on the FBGs. All but two samples were produced without stripping the native acrylate recoating of the FBGs. In Section 3.1, details of the procedure and substrates used for the production of the samples are reported. The full set of samples was not fully planned in advance. The results achieved with the sample initially produced provided motivations that addressed the production procedure of the other ones in an iterative manner.

The chitosan coating was deposited as a solution prepared by dissolving Low Molecular Weight (LMW) chitosan (Acros Organics, catalogue n. ACRO34905) with a concentration of 5% wt. in 1% aqueous acetic acid solution. Since the chitosan solution is quite viscous and traps air bubbles into the coating film, a de-gassing process before the deposition had to be adopted. The chitosan gel was deposited on the FBGs and dried at room temperature. All chemicals were reagent grade (Sigma-Aldrich) and were used without any purification or processing.

### 2.3. Measurements Set-Up

Data acquisition was done with a commercial FBG interrogation system (FS22, HBM FiberSensing; resolution = 1.0 pm, sampling frequency = 1 Hz). A thermo-hygrometer (EasyLog, EL-USB_2, resolution 1% RH, 1 °C) was used to have reference RH and temperature measurement. Due to the cross-sensitivity of FBGs to strain and temperature, a bare FBG without coating was used as a temperature compensation sensor (standard procedure in FBG sensing [36]).

**Figure 1:**
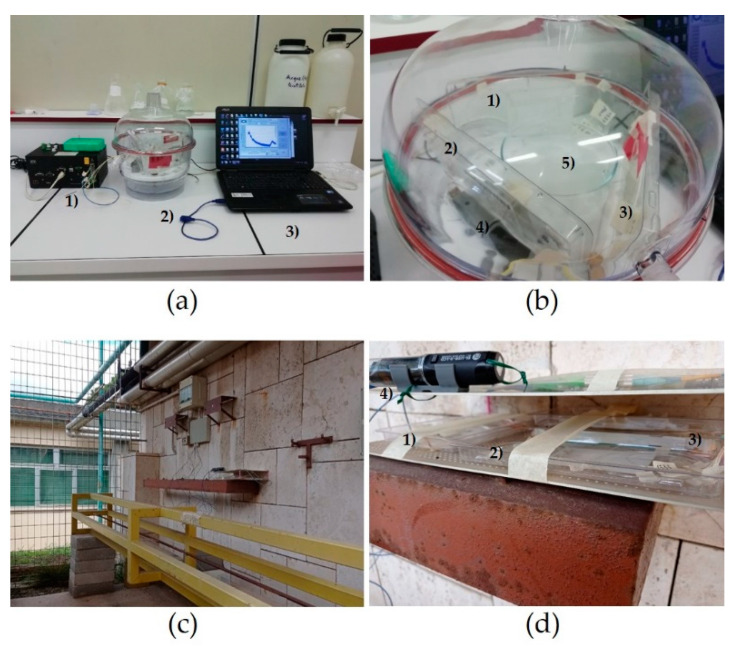
Experimental set-ups. (**a**) Measurements with the saturated salt solutions: (1) FBG interrogation system; (2) humidity chamber; (3) laptop. (**b**) Close view of the humidity chamber: (1–2–3) samples; (4) thermo-hygrometer; (5) salt saturated solution. (**c**) Location for the measurements in the open air. (**d**) Close view of the tray with the samples: (1–2–3) samples; (4) thermo-hygrometer.

Measurements were carried out in an airtight humidity-controlled chamber (Figure 1a,b) where saturated chemical salt solutions [38] were used to modify humidity. The use of chemical saturated salt solutions is a widely adopted method in humidity calibration and provides an effective method to measure sensitivity. RH was modified back and forth between 29% and 84% for a few cycles, at constant room temperature. Three different kinds of chemical saturated salt solutions were prepared to generate RH conditions at about 29% RH (CaCl_2_·6 H_2_O), 51% RH (Ca(NO_3_)_2_·6 H_2_O), and 84% RH (KCl). The measurements with the three reference RH points have shown a linear trend for all samples, as reported in Section 3.2. For the scope of this work, it was not of interest to investigate further the resulted linearity, thus no additional reference RH points were tested. RH and temperature in the airtight chamber were measured by the reference thermo-hygrometer.

Tests were carried out in the open air (Figure 1c,d) with measurement campaigns of several days repeated with various climate conditions.

## 3. Results and Discussion

### 3.1. Design and Production Procedure of Samples

Chitosan was chosen as a humidity-sensing polymer because it has a large swelling capacity in water while maintaining integrity by regulating the moisture absorption and preventing the swelling from occurring too violently [31]. On the other hand, it has a strong filming performance that prevents it from forming a regular coaxial coating around the fibre. After a few tests, it became clear that the viscosity of the gel is a critical parameter for obtaining an efficient embedding of the fibre in the chitosan film. The viscosity of the chitosan solution can be controlled by simply varying the concentration of the acetic acid. The viscosity of the gel decreases with increasing acid concentration due to the repulsion between the electrostatic chains induced by the presence of acidic protons. A higher viscosity allows better shape retention during the processes of solvent evaporation and film forming. First of all, the chitosan embedding performance was tested using optical fibres with no FBG (dummy samples). The solutions of chitosan with High and Low Molecular Weight (HMW, LMW), with different concentrations (namely, 2% and 5% wt.), and in different solvents (namely, aqueous solutions of acetic acid at 1%, 2%, 4% wt.) were used, too. The results were qualitatively compared by evaluating the morphology of the chitosan layer by optical microscopy and the adhesion of the layer on the fibre by peeling it off with soft tissue. The best results were obtained with a solution of LMW chitosan with a concentration of 5% wt. in 1% aqueous acetic acid. This solution was selected for the production of the samples.

**Figure 2:**
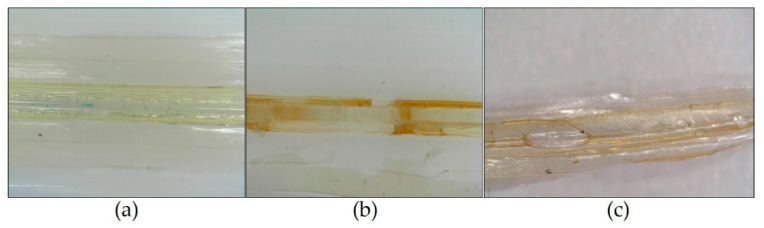
Microscopic photos of three samples produced with different chitosan solutions: (**a**) LMW chitosan, 5% wt. in 1% aqueous acetic acid; (**b**) LMW chitosan, 5% wt. in 2% aqueous acetic acid; (**c**) HMW chitosan, 5% wt. in 1% aqueous acetic acid.

Figure 2 shows the microscopic photos of three samples produced with different solutions: (a) LMW chitosan, 5% wt. in 1% aqueous acetic acid; (b) LMW chitosan, 5% wt. in 2% aqueous acetic acid; (c) HMW chitosan, 5% wt. in 1% aqueous acetic acid. In the pictures, the different morphology of the coatings can be seen. The best result is in Figure 2a, which shows the homogenous coating obtained with the solution selected for the production of the FBG samples listed in Table 1. 

As shown in Table 1, six samples were produced adopting different designs, materials, and preparation of the optical fibre. The samples are listed from top to bottom in the chronological production order. The values of the sensitivity S_RH_, evaluated with the measurements reported in Section 3.1, show the improvements achieved thanks to the adopted iterative production procedure, as explained below.

**Figure 3:**
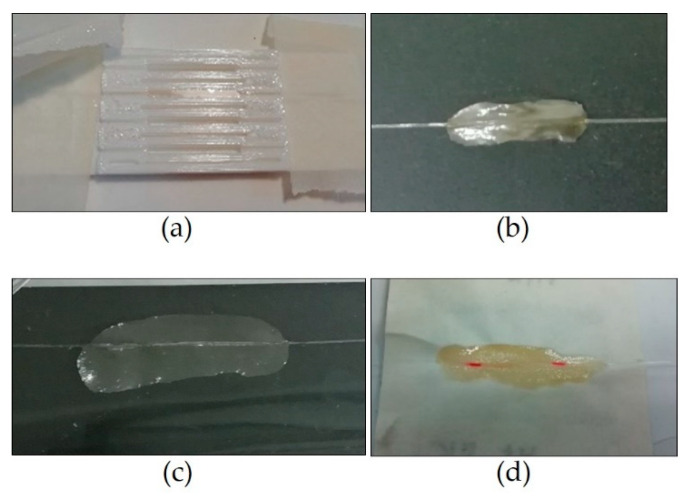
Photos of four samples. (**a**) FBG-PLA in the mould; (**b**) FBG-ACET detached from the acetate sheet; (**c**) FBG-PVC on the PVC layer; (**d**) FBG-FP on the filter paper.

The first produced sample, named FBG-PLA, was made in a mould of polylactide (PLA) fabricated by a 3 D printer with forms 0.5 × 0.5 × 3 cm (Figure 3a, the mould has four forms). The sample was produced according to the procedure described in [28] to produce sensors with agar coating. The sample consists of a block of chitosan surrounding the FBG. As discussed in the next section, sample FBG-PLA resulted to have poor reversibility. The poor reversibility was ascribed to the thickness of the chitosan coating, but the film-forming performance of the chitosan did not allow the successful production of thinner blocks. Block production was thus dismissed, with the intent to produce FBG RH sensors embedded in a thin layer, taking advantage of the film forming performance of the chitosan and the possibility to control the viscosity of the chitosan gel.

The production of a thin sample was tested on a plane substrate. The sample named FBG-ACET was produced on a flexible acetate sheet, in order to form a film that was easily detachable from the substrate (Figure 3b, the chitosan film embedding the fibre can be seen). As discussed in the next section, the tests with sample FBG-ACET confirmed that better performance can be obtained with thin chitosan coating, but the sample resulted to be fragile and unprotected from accidental strain. To solve that issue, the sample named FBG-PVC was produced on a thin and rigid polyvinyl chloride (PVC) substrate, with the intent to form a film attached to the substrate (Figure 3c, PVC substrate is transparent, a black background enhances the visibility of the sample). The production of the sensor on a somehow rigid layer, as expected with the PVC substrate, was intended to overcome the cross-sensitivity of the FBG to accidental strain due to direct mechanical disturbances.

As discussed in the next section, sample FBG-PVC resulted to have low sensitivity to RH. The sample named FBG-PVC-S was produced with the intent to improve the sensitivity. The native acrylate recoating of the FBG was stripped before processing the bare fibre with the same procedure adopted for sample FBG-PVC. In fact, the acrylate recoating can act as a soft layer that reduces the strain transfer from the swelling chitosan to the Bragg grating. Although the sensitivity was improved, critical considerations arose about the sensor being produced on a not permeable layer.

A new design was tested adopting qualitative filter paper (FP) (Whatman^®^ qualitative filter paper, Grade 1) as a substrate. The substrate was chosen with two intents: to have a texture-type layer to improve the adhesion of the chitosan on it; to improve the permeability of the substrate, useful for monitoring masonry walls and stones. Two samples were produced, stripping and not stripping the native acrylate recoating, named FBG-FP-S and FBG-FP, respectively. As discussed in the next section, this design proved to have the best performance. A rigid frame was used to hold the paper and protect the sensor from accidental strain (Figure 3d, the holding frame is under the paper).

**Figure 4:**
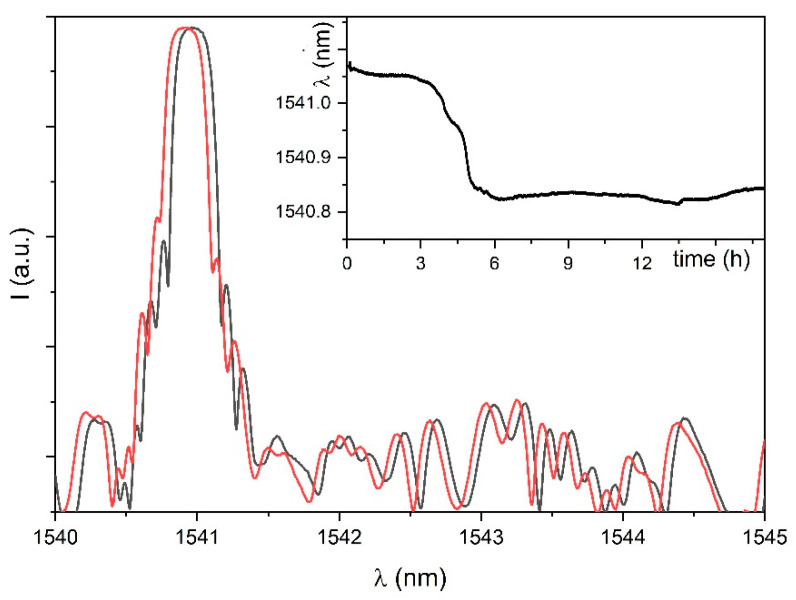
Spectra of sample FBG-PVC before (black) and after (red) the film forming process. In the inset: the time history of λ_B_ during the film forming process.

For each sample, the effect of the coating deposition process on the spectra of the FBG was monitored. Spectra did not show relevant broadening or distortion of the peaks, which is an important feature to control, as it would negatively affect the correct detection and processing of the signal with commercial FBG interrogation systems. In fact, the spectra of the commercial FBG sensors have a main bell-shaped component whose maxima is at λ_B_ (Equation (1)), and commercial FBG interrogation systems operate with peak-search algorithms that assume that kind of spectra. All samples showed a shift of the λ_B_ toward shorter wavelengths, as expected for the compression that the FBG sensor suffers because of the polymer film forming process. As an example, the spectra of sample FBG-PVC before and after the film forming process are reported in Figure 4. In the figure, the inset shows the time history of λ_B_ during the film forming process.

### 3.2. Sensors Characterization and Calibration

**Figure 5:**
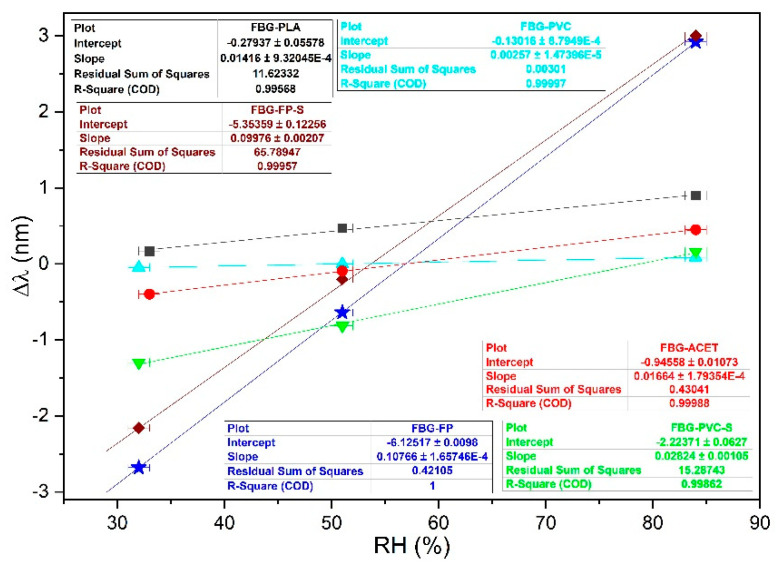
Experimental data (dots) and linear fits (lines) providing the sensitivities listed in Table 1: FBG-PLA (square); FBG-ACET (dot); FBG-PVC (up-triangle); FBG-PVC-S (down-triangle); FBG-FP (star); FBG-FP-S (diamond).

Samples were tested in an airtight humidity-controlled chamber by use of saturated salt solutions at room temperature. RH calibration was done, as explained in Section 2.2, by multiple-step changes of RH, repeating the procedure to attest the reproducibility. Samples showed a linear response. In Figure 5 data for all the samples are reported. For example, the linear fit of the data for sample FBG-FP yields a slope of *b* = 0.107 ± 0.001 nm%RH^−1^, resulting in S_RH_ = (70.4 ± 0.5) 10^−6^ %RH^−1^ (equation 4, λ_B_ = 1529 nm). The sensitivity of each sample is reported in Table 1.

Figure 5 shows that the samples produced on filter paper have the highest sensitivities. To verify that the good performance of sample FBG-FP and sample FBG-FP-S was due to the design and the improved contribution of the chitosan coating, a dummy sample was tested. The dummy sample was made with a FBG without chitosan coating and stuck on the filter paper by a non-hygroscopic glue for structural applications. Tests were thus worked out to check if the filter paper by itself was responsible for the swelling detected by the FBG. Results show that only the expected intrinsic response of the FBG to temperature occurs, with no evident response to humidity.

Figure 5 also shows that sample FBG-PLA, sample FBG-ACET, and sample FBG-PVC-S have similar sensitivities, lower than the sensitivity of sample FBG-FP. Sample FBG-PVC has very low sensitivity, so low that the response of the sample to RH merged with the signal due to the response to temperature. For sample FBG-PVC, a possible relationship with the dew point was investigated and a good correlation was found.

### 3.3. Test in the Environment

Samples were tested in the environment. The tests for every sample lasted several days and samples were re-tested at different seasons of the year. Samples were produced in sequence and the results achieved with the first ones were used to address the production of the following ones. A unique final comparative test with all samples was not done because of the limits of the available FBG interrogation system. For every sample, at least two tests were done at different seasons of the year to have a more significant range of climatic conditions.

**Figure 6:**
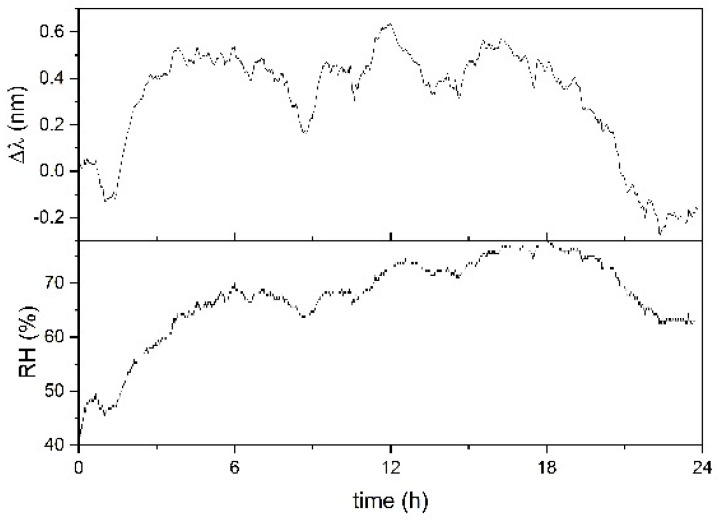
Response of sample FBG-PLA for 24 h in December. RH value was measured by the reference thermo-hygrometer.

**Figure 7:**
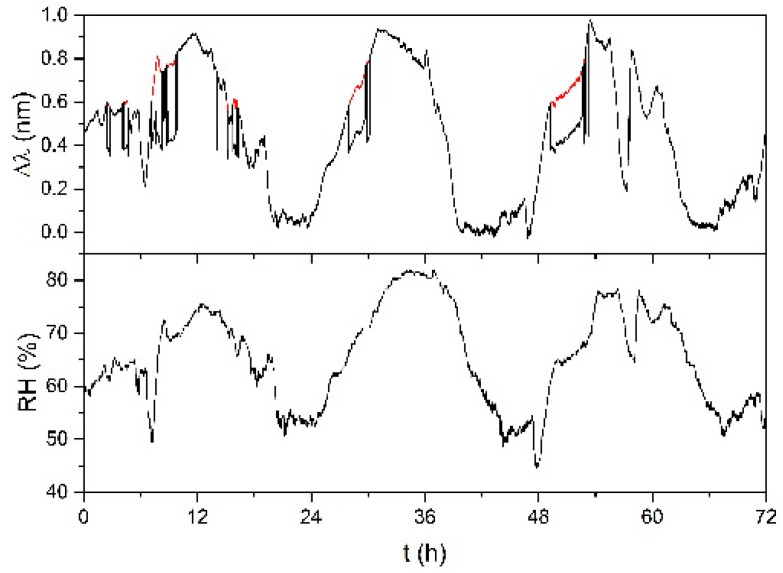
Response of sample FBG-ACET for three days in May (black); red sections are discussed in the text. RH value was measured by the reference thermo-hygrometer.

**Figure 8:**
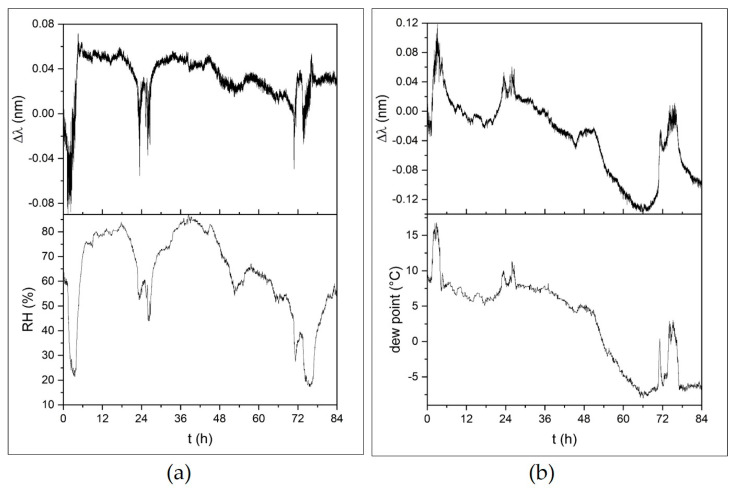
Response of sample FBG-PVC for three days in May: (**a**) raw signal, (**b**) temperature-compensated signal. RH and the dew point were measured by the reference thermo-hygrometer.

**Figure 9:**
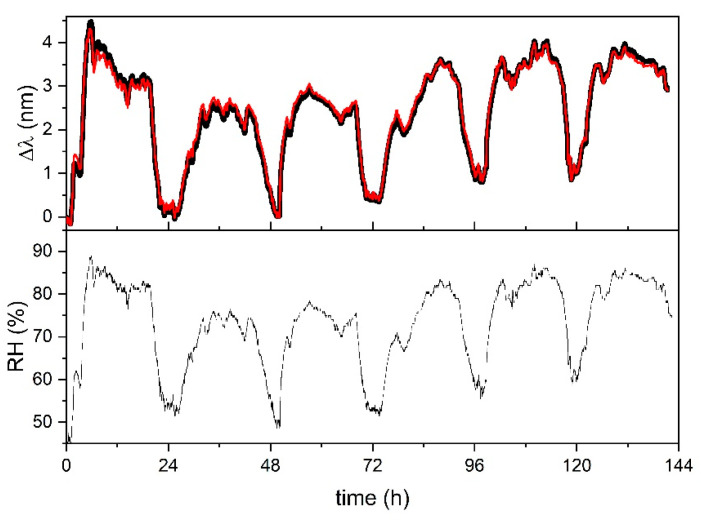
Response of sample FBG-FP (black) and sample FBG-FP-S (red) for six days in October. RH value was measured by the reference thermo-hygrometer.

**Figure 10:**
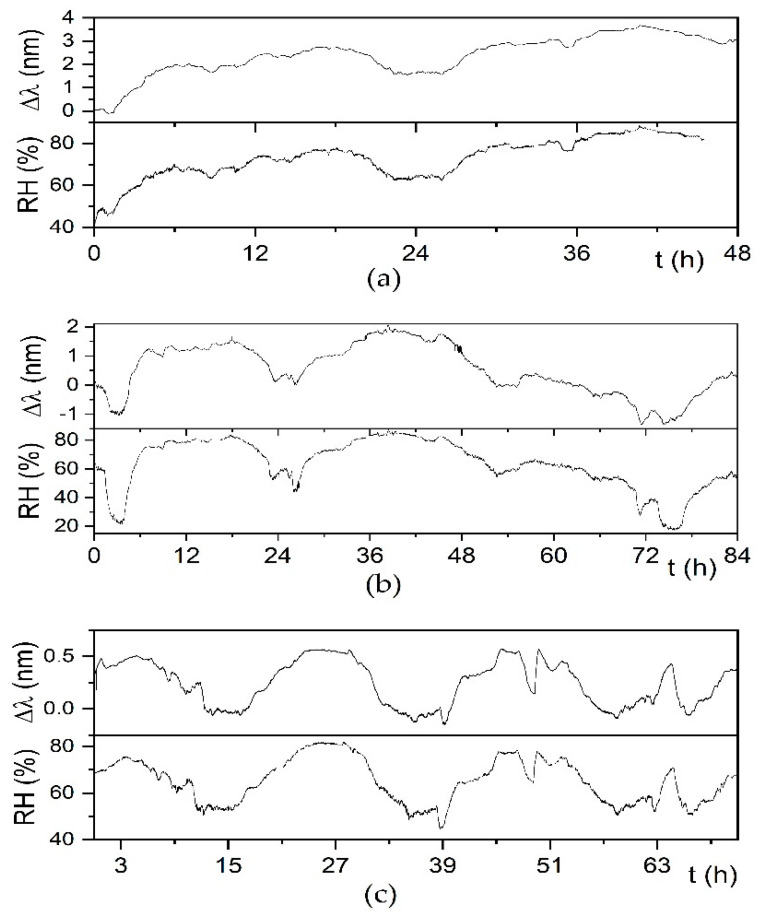
Response of sample FBG-FP in different periods of the year: (**a**) for two days in December; (**b**) for three days in February; (**c**) for three days in May. RH value was measured by the reference thermo-hygrometer.

The performance of each sample was evaluated with respect to the response to RH, as measured by the reference thermo-hygrometer. Representative examples of the observed performance of all samples are shown in Figure 6, Figure 7, Figure 8, Figure 9 and Figure 10. Each figure shows the time history of one sample and the time history of the reference thermo-hygrometer. 

In Figure 6, a time history of sample FBG-PLA is shown. The measurement was done in December, with RH variation from 40% to 75%. It can be seen that the sample does not have a good performance. The response to RH has some evident violent swellings and de-swellings that alter the correlation. The violence of the swelling is evident in the time interval 0–6 h, where the increase of the signal occurs with a rise much steeper than the RH rise. A similar consideration applies to the de-swelling occurring in the time interval 18–24 h, which also shows that the process ends with a final excessive de-swelling. An unreliable response appears in the time interval 3–6 h; the sample does not follow the slowly increasing trend of RH but shows large peaks when the small RH temporary peaks occur. The unreliable response of sample FBG-PLA was ascribed to an excessive thickness of its chitosan coating, with consequent excessive adsorption and retention of humidity. A sensor with a thin coating was thus tested, producing sample FBG-ACET with a design based on the embedding of the FBG in a thin film of chitosan, as described in Section 3.1.

In Figure 7, a time history of sample FBG-ACET is shown. The measurement was done in May, with RH variation from 45% to 80% (similar to the variation shown in Figure 6 for FBG-PLA). The response of the sample is drawn in black, with some red sections overdrawn. Each overdrawn red section is obtained by a shift of the corresponding (same time interval) black section, with some continuous red section being the result of the shift of multiple sub-sections (evident at time ≈ 8 h). If the red sections are considered instead of their corresponding black sections, the sample shows a good response. The introduction of the red sections corresponds to the compensation of some accidental strain that affected the sample. In fact, the sample has no structural support; thus, it is subject to mechanical disturbances that can cause a response much larger than the one caused by the chitosan swelling. Occurred disturbances were due to natural events (irregular wind, as at time ≈ 8 h) and human activities (operator handling other sensors and devices, mainly step-like). With the red sections, the improvement of the performance of sample FBG-ACET with respect to sample FBG-PLA is evident. The response to RH is good with no evidence of saturation and no evidence of missed recovery. The improvement of the results obtained with sample FBG-ACET confirmed the validity of its design. Nonetheless, having the FBG completely unprotected from accidental strain was considered a severe issue. Sample FBG-PVC was thus produced with the chitosan filmed on a rigid substrate, as described in Section 3.1.

Sample FBG-PVC showed a low sensitivity, which was ascribed to a reduction of the swelling of the filmed chitosan owing to the fibre being glued to a rigid substrate and the chitosan being filmed over a not permeable substrate. In order to increase the sensitivity of the sensor, sample FBG-PVC-S was produced, as discussed in Section 3.1. During the environment test of FBG-PVC, the sample did not show a good response to RH. On the contrary, the sample showed a good response of the raw (not compensated for temperature) signal to the dew point. Dew point is much preferred in some applications, for example in meteorology, as it provides a better “absolute” measurement of water vapour content than RH measurements, which may fluctuate with temperature [39]. In Figure 8, the time history of three days in February with RH variation from 25% to 85% is reported: (a) comparison of the temperature-compensated signal with RH; (b) comparison of the raw signal with the dew point value measured by the reference thermo-hygrometer.

The good response of sample FBG-PVC to the dew point suggests the possible development of sensors for such parameters based on the FBG technique and suggests future work to test FBG-PVC-S for that. As for FBG RH sensors, the design based on a not permeable substrate was considered to be inefficient and sample FBG-PVC-S was not tested in the environment. Sample FBG-FP and sample FBG-FP-S were thus produced with the chitosan filmed on a permeable substrate, as described in Section 3.1.

Sample FBG-FP and sample FBG-FP-S were produced and tested in parallel. In Figure 9, a time history of the two samples is shown. The measurement was done in October, with RH variation from 45% to 90% (similar to the variation that occurred in the tests of the other samples). In the calibration tests, as reported in Section 3.2, a slightly larger sensitivity resulted for sample FBG-FP, but the response of the two samples in the environment are very similar and the two plots practically overlap. In the time intervals 0–24 h and 108–144 h, the expected slightly larger excursion occurs for sample FBG-FP, but it is not a steady feature. As a possible explanation, the occurrence of a slight and unstable RH difference at the locations of the two samples, due to uncontrolled air currents in the open air, was assumed. For both samples, the response to RH is good with no evidence of saturation and no evidence of missed recovery.

Figure 10 shows the response of sample FBG-FP in different periods of the year: (a) December; (b) February; (c) May. The three plots, of different length to provide evidence of the quality of the response with an expanded view in shorter intervals and with a general view in longer intervals, confirm the good response in the long term and different real climatic conditions.

## 4. Conclusions

In this paper, the use of chitosan as a possible swelling coating for the production of FBG RH sensors is demonstrated. Chitosan was chosen because of its large swelling capacity in water and structural ability to regulate moisture absorption, which prevented uncontrolled swelling and assured more stable behaviour. Different sensor designs were tested to address the filming performance of chitosan and the possibility to have it in a gel form in which viscosity can be easily controlled. Experimental tests show that the best results are obtained by coating deposition with the FBG laying on filter paper. The design provides a sensor well suited for monitoring humidity both in the air and on the surface of the materials. The latter configuration is of specific interest for planned activity in the field of cultural heritage, in particular for the conservation of stones and masonry walls in architectural complexes. The sensor proved to have a linear response to humidity in the 29%–95% RH range. Future work is necessary to better characterize the specifications of the sensor and to assess the stability of its features in the long term. That work shall be done with the production of multiple nominally identical sensors to verify the full control and reproducibility of the production procedure.

## Figures and Tables

**Table 1:** List of the samples.

Sample Name	Material–Design–Fibre Preparation		S_RH_ × 10^6^
	Substrate/Mould	Stripping	%RH^−1^
FBG-PLA	polylactide/mould	No	9.2 ± 3
FBG-ACET	acetate/substrate	No	10.8 ± 3
FBG-PVC	polyvinyl chloride/substrate	No	1.7 ± 0.5
FBG-PVC-S	polyvinyl chloride/substrate	Yes	18.2 ± 0.7
FBG-FP	filter paper/substrate	No	70.4 ± 0.5
FBG-FP-S	filter paper/substrate	Yes	64.7 ± 5
